# Guidance for family about comfort care in dementia: a comparison of an educational booklet adopted in six jurisdictions over a 15 year timespan

**DOI:** 10.1186/s12904-022-00962-z

**Published:** 2022-05-17

**Authors:** Laura Bavelaar, Adrienne McCann, Nicola Cornally, Irene Hartigan, Sharon Kaasalainen, Hana Vankova, Paola Di Giulio, Ladislav Volicer, Marcel Arcand, Jenny T. van der Steen, Kevin Brazil, the mySupport study group

**Affiliations:** 1 Department of Public Health and Primary Care, Leiden University Medical Center, 9600, Hippocratespad 21, 2300 RC Leiden, the Netherlands; 2 Innovation Value Institute, Maynooth University/Age Friendly Ireland, Maynooth, Ireland; 3 Catherine McAuley School of Nursing and Midwifery, University College Cork, Cork, Ireland; 4 School of Nursing, McMaster University, Hamilton, Ontario Canada; 5 Center for Palliative Care, Prague, and Third Faculty of Medicine, Charles University, Prague, Czech Republic; 6 Department of Sciences of Public Health and Pediatrics, Turin University, Milan, Italy; 7 School of Aging Studies, University of South Florida, Tampa, FL USA; 8 Department of Family Medicine, University of Sherbrooke, Sherbrooke, QC Canada; 9 Department of Primary and Community Care, Radboud university medical center, Nijmegen, the Netherlands; 10 School of Nursing and Midwifery, Queen’s University Belfast, Belfast, Northern Ireland UK

**Keywords:** Decision aid, Dementia, Education, End of life, Family caregivers, Nursing homes, Palliative care

## Abstract

**Background:**

To support family caregivers of people with dementia in end-of-life decision making, a family booklet on comfort care has been adapted and adopted by several European jurisdictions since the original publication in Canada in 2005.

**Methods:**

We analyzed and compared the adaptations to the family booklets used in Canada, the Czech Republic, Italy, the Netherlands, the UK and Ireland that were made up to 2021. Qualitative content analysis was used to create a typology of changes to the original booklet. Interviews with the teams that adapted the booklets contributed to methodological triangulation. Further, using an established framework, we assessed whether the contents of the booklets addressed all domains relevant to optimal palliative dementia care.

**Results:**

The booklets differed in the types of treatment addressed, in particular tube feeding, euthanasia, and spiritual care. There was also variability in the extent to which medical details were provided, an emphasis on previously expressed wishes in medical decision making, addressing of treatment dilemmas at the end of life, the tone of the messages (indirect or explicit) and the discussion of prognosis (as more or less positive), and the involvement of various healthcare professionals and family caregivers in care. All booklets addressed all domains of palliative dementia care.

**Conclusions:**

We identified core elements in providing information on end-of-life care to family caregivers of people with dementia as related to optimal palliative care in dementia. Additionally, local adaptations and updates are required to account for socio-cultural, clinical, and legal differences which may also change over time. These results may inform development of educational and advance care planning materials for different contexts.

**Supplementary Information:**

The online version contains supplementary material available at 10.1186/s12904-022-00962-z.

## Background

Alzheimer’s disease and other neurodegenerative diseases causing dementia are progressive and life-limiting illnesses, characterized by symptoms such as behavioral symptoms and cognitive decline and, in later stages, food and fluid intake problems [1]. Therefore, a palliative care approach is appropriate. When dementia progresses to more severe stages, goals of care may shift from prolongation of life to maximizing comfort [2]. In order to provide person-centered care, these care goals should reflect individual wishes [2]. Due to cognitive impairment, family caregivers advocate for their relatives with dementia in conversations about goals of care and decision-making [3, 4]. This is a difficult task for which many family caregivers feel ill-prepared. They may not be aware of the terminal course of dementia and may lack knowledge about palliative care [5]. Such information may be crucial as nursing staff have reported higher comfort in dying for people with dementia whose family are aware of the disease prognosis, in part because their healthcare professionals are being able to provide better end-of-life care [6].

The World Health Organisation (WHO) urges to assist family caregivers with information about dementia and palliative care [1]. In Canada in 2005 the Comfort Care Booklet [7], a guide for caregivers of people with dementia, was developed with this aim and has been adopted by the WHO as an example of good practice [1]. This informational booklet informs family caregivers regarding the course of dementia and palliative care options. The booklet intends to help family caregivers understand that a palliative approach to care is appropriate and does not imply that “nothing can be done”. Instead, a palliative approach to care can be considered a ‘low-tech’, but ‘high-touch’ approach [8]. Retaining its core, the booklet has been translated and adapted for use by healthcare professionals and researchers in several European jurisdictions since 2005: Italy (2008) [9], the Netherlands (2011) [10], the Czech Republic (2017) [11], Ireland (2020) [12] and the UK (2021) [13]. Further, in 2021, a new edition of this Canadian Booklet was developed [14].

Cross-national work about the Japanese, Italian, Dutch and original Canadian version showed that solely translating the information does not suffice. Adaptations to the local context are necessary for the booklets to be applicable and acceptable [15]. In addition, it is important that educational information is based on current evidence-based practice [16], such as the recommendations by the European Association for Palliative Care (EAPC) about optimal palliative dementia care in older people first issued in 2013 [2]. Furthermore, developments in evidence and evolving public perception require that information should be reviewed regularly to remain up to date [17, 18].

In this paper, we aim to provide guidance about the contents of informational booklets for family caregivers about dementia and palliative care, considering (i) transnational legal and socio-cultural differences and developments over time, plus (ii) evidence and expert consensus-based recommendations regarding palliative dementia care. We compared informational booklets from six jurisdictions to determine key topics and we performed content analysis to highlight contextual differences. The EAPC recommendations for optimal palliative dementia care [2] were mapped onto the contents of the booklets.

## Methods

This qualitative descriptive study [18] was conducted as part of an international multiple case study called *mySupport study*, which involves Canada, the Czech Republic, Italy, the Netherlands, Ireland and the UK. The *mySupport study* aims to support family caregivers of nursing home residents with advanced dementia in decision making about end-of-life care [19]. In addition to training staff in conducting family care conferences, family caregivers are provided with information about the progression of dementia and end-of-life care for nursing home residents with dementia via the Comfort Care Booklet [20].

### Comparison of content

To compare the booklets’ contents transnationally, we took a deductive approach to identify (i) key topics of the Comfort Care Booklets, as they are presented in all the booklets, and (ii) topics that require adaptation to the specific socio-cultural, legal or temporal context, as they differ between the booklets.

First, the Czech, Dutch and Italian booklets were translated back to English. Next, two researchers (LB and JTvdS) read all the booklets thoroughly and compared the contents of all the booklets with the original Canadian booklet. Differences were marked and listed in a matrix. Then, semi-structured interviews with the editors of the local booklets provided input for methodological triangulation verifying the comparison exercise for completeness [21, 22]. A comparison between the original Canadian, the Italian and the Dutch version of the booklet has been reported previously [15]. Therefore, LB and JTvdS selected the editors of the Czech, UK, Irish and updated Canadian versions of the booklets for an interview. The interview guide asked about topics that were added, deleted, or revised compared with the original Canadian booklet, and about the stakeholders involved in adapting the booklet. Interviews were transcribed, summarized and fed back to the developers for member checking, clarification and elaboration.

Finally, qualitative directed content analysis was performed on the identified differences of each booklet compared with the original Canadian booklet [23]. The tabulated differences were first read repeatedly to create familiarity with the data. Then, the differences were assorted into categories that were informed by the literature [15, 24]. Differences that could not be coded in this manner were identified and were assorted in an additional category or labeled as a subcategory of the existing categories. Next, the categories were reviewed and finalized.

### Quality appraisal

A transnational quality appraisal was performed using a deductive approach to identify (i) if key topics according to evidence and expert consensus-based guidelines were represented in the Comfort Care Booklets (cf. [25]), and (ii) if this differed for the various booklets. To facilitate a comparison between the booklets (aim ii), the quality appraisal was performed with a single international (EAPC) framework, rather than multiple national guidelines on palliative dementia care.

To support methodological validity, AM and LB first developed a protocol with accompanying grid for mapping the content of the booklets against the recommendations presented in the EAPC framework [2, 26] as depicted in Box 1 (Additional file 1). The mapping did not include Domain 10: Education of the healthcare team and Domain 11: Societal and ethical issues, as these domains are not expected to be *explicitly* stated in the booklets—although ethical and moral challenges may be considered within the booklets. For each booklet, this protocol was shared with a researcher fluent in the local language and who was familiar with the content of the local booklet. The outcomes of the final consensus mappings were entered in a grid to facilitate comparison across the booklets.

## Results

### Comparison of content

When comparing the contents of the booklets, two versions appeared: booklets that were based on the 2005 Canadian Comfort Care Booklet (the 2021 Canadian booklet, the Czech booklet, the Italian booklet, the Dutch booklet and the UK booklet) and booklets that were based on the 2017 UK booklet (the Irish booklet and sections of the 2021 Canadian booklet), see Table 1 (Additional file 2). The booklets that were based on the UK booklet thus had not used the original Canadian booklet as the starting point, but were based on the UK booklet -retaining the adaptions that were made in the UK booklet. Both healthcare professionals and family caregivers were involved in evaluating the contents of the booklets and the adaptations. This involvement ranged from participation in a study leading up to the development of the booklet [27] (indirect involvement), to “collaboration and co-production” which entailed team membership and contributing to key decisions [28]. Healthcare professionals largely influenced the content revisions. Revisions often concerned the local legal frameworks and shared decision making practice.

The key topics that were present in all the booklets were the progression of dementia and possible complications such as eating difficulties and infections, the decision-making process about treatment options at the end of life, palliative care and managing symptoms such as pain, breathing problems and anxiety, the dying process and common emotions and procedures after death. These key topics were retained from the original Canadian Comfort Care Booklet.

We arranged the textual revisions of the booklets compared with the original texts in six categories: 1.Typology of treatments and symptoms at the end of life, 2. Patient and family rights and wishes, 3. Typology of decisions at the end of life, 4. Indirect or explicit messages, 5. More or less positive about prognosis, and 6. Relationship between healthcare professionals and family caregivers.

#### Typology of treatments and symptoms at the end of life

The booklets differed in the treatment options that they described, and the level of medical detail that was provided about symptoms and treatments. Three topics related to treatment differed the greatest between the booklets: artificial nutrition, life-terminating or life-limiting treatments and spiritual care. Whereas all booklets discussed eating difficulties in advanced dementia, the UK booklet did not contain information about tube feeding or any objections to it (Table 2 (Additional file 3), quote A). Instead, extensive information about oral hygiene was provided and this was also included in the Irish and updated Canadian booklet. The Czech booklet provided detailed information about alternative feeding and food options to address eating difficulties. The Czech and Italian booklet provided more medical information about the complications of artificial nutrition during the dying process than the other booklets. This was included because it was considered difficult to convince family caregivers not to start artificial feeding at end of life. Regarding information about euthanasia, this was not included in the Irish and UK booklets, while more elaborate information was provided in the Dutch booklet and updated information in the Canadian booklet. The Czech and Italian booklets mentioned euthanasia only to state that it is not a viable option. The Dutch and Canadian booklets had included additional information about palliative sedation. The UK, Irish and updated Canadian booklet offered information on spiritual care, which was not included in the Czech, Dutch and Italian booklets.

The extent to which the booklets offered detail about medical issues varied, and this may relate to difference in whether healthcare professionals or family caregivers were the most influential in the revision process (see also Table 1 (Additional file 2) Stakeholder Involvement). The Czech, Dutch and Irish booklets contained the most information regarding medications and physical health and the UK booklet the least. For example, the Czech, Dutch and Irish booklet provided detailed information about the breathing pattern during dying or extra information about pain management options. In contrast, the UK booklet did not speak about the medical complications that could arise after hospitalization when addressing why transfer to hospital may not be appropriate, while the other booklets did.

#### Patient and family rights and wishes

The varying legal systems of the jurisdictions were apparent in diverging emphasis on patient rights and wishes between the booklets. The Czech and Irish booklets stood out the most in this respect. The Czech booklet included an entire section about living wills and legally binding wishes to refuse care, driven by the Health Services Act, No. 372/2011 Coll [29]. Emphasis was placed on acting in accordance with living wills throughout the booklet. The Irish booklet contained several sections that asked family caregivers to think about previous wishes of the person with dementia, to ensure that any decision making is aligned to the person’s previously stated will and preference. Reference was made to Ireland’s Assisted Decision-Making (Capacity) Act 2015 [30]. The UK booklet and Dutch booklet only referred to patient wishes for specific treatment decisions, such as CPR and the use of antibiotics, respectively.

The Italian booklet clearly stated that family members provide only information for the decision-making process. This mirrors the updated Canadian booklet, that had adapted the terminology to current legislation regarding shared decision making (Table 2 (Additional file 3), quote B). That is: the family caregiver was referred to as “the patient’s legal representative” instead of “the mandatory” (a term formerly used to indicate a representative by law) and a statement was added about variations in provincial laws across the country regarding the role and rights of this person. The updated Canadian booklet, and the Dutch booklet, introduced family wishes only in the section on providing the last care after death.

Further, no mentioning of settling disagreements in court was made in the Czech, Italian and Dutch booklets. The Czech booklet also did not refer to the assistance of ethics committees. A final difference between the booklets was that only the Dutch and Canadian booklets contained information about the legal requirements for life terminating treatments.

#### Typology of decisions at the end of life

A similar category of differences between the booklets related to the description of end-of-life decision making. The Irish and Czech booklets emphasized best interest decision making, involving the family caregiver. The UK booklet underscored the responsibility of the clinician or medical team to facilitate this.

When describing considerations for decisions or treatment, the booklets varied in whether they stated the underlying dilemma. The UK and Italian booklets often did not include the dilemma. For example, they did not refer to pneumonia as “the older person’s best friend” (an argument against curative treatment of pneumonia, Table 2 (Additional file 3), quote C). Dilemmas were sometimes emphasized in the Czech booklet, for instance by adding the statement “even at the cost of reduced comfort” which suggests curative treatment can be incongruent with comfort care. The moral acceptability of treatment decisions was, at some places, omitted from the Dutch and Irish booklet. The decision to increase doses of morphine at the end of life to reduce suffering was therefore more a medical than a moral decision, for instance.

#### Indirect or explicit messages

An evident difference between the booklets was their layout. While the Dutch and original Canadian booklets contained images of moments of caring, the UK booklet contained images of nature. The Italian, Czech, Irish and updated Canadian booklets were in the middle of this spectrum and showed images of their local nursing home contexts. In addition, the Irish booklet contained images of nature.

Differences between explicit messages or more softened, indirect messages were also found in the text in terms of style. The UK booklet used more softened language, for example comparing breathing problems to asthma. This booklet also spoke about nausea or discomfort, similar to the Italian and Irish booklet. The Czech, Dutch and Canadian booklets instead mentioned vomiting and pain. The Czech booklet typified useless or harmful treatment in the last days or hours of terminal illness as “dysthanasia”, detained death, and mentioned more confrontational treatment details.

All booklets considered dementia as a terminal condition, but some booklets were more explicit about this. The Czech booklet further contained explicit statements about the non-curable and terminal nature of the disease causing dementia syndrome. Also the Irish booklet explicitly mentioned the dying phase several times. The Dutch booklet clearly related not eating and drinking to the dying phase (Table 2 (Additional file 3), quote D).

All booklets recommended a palliative care approach based on physical and psychological comfort; the Canadian and Italian booklets concluded with the statement that “That’s because the majority of people perceive that advanced and prolonged dementia is worse than death”. This statement was not incorporated in the other booklets.

#### More or less positive about prognosis

There was some variation within and between the booklets regarding the description of the prognosis. The Czech booklet started with the limited life expectancy and cause of death in the introduction (Table 2 (Additional file 3), quote E) and therefore appeared less positive about the prognosis compared with the other booklets. The symptoms that were described in this booklet were mainly possible causes of death, as was the case for the Canadian, Dutch and Irish booklets. The UK and Irish booklets had additionally included symptoms related to activities of daily living, describing less severe stages of dementia. This encompassed a more holistic tone and upstream approach regarding prognosis than referring only to symptoms around the end of life.

The Czech booklet was less positive about prognosis throughout the booklet, for example stating how certain treatments may not be tolerated by the person with dementia. The more positive tone about prognosis of the UK booklet was also present throughout, for example by not stating some negative consequences of treatments. The Dutch booklet was more positive about prognosis in some sections: a maximum estimate of survival was provided for people who do not eat (instead of a time window that included a shorter time estimate). However, in other sections, the Dutch booklet was less positive about prognosis: it included the statement that the “final stage can be long and exhausting”.

#### Relationship between healthcare professionals and family caregivers

Two booklets stood out regarding the relationship between healthcare professionals and family caregivers: the updated Canadian and Irish booklet. Both had included information about family involvement in care and this was particularly present throughout the Irish booklet (Table 2 (Additional file 3), quote F). The other booklets did not include this information, apart from sitting in at the end of life. The Irish booklet additionally referred to several healthcare disciplines throughout the booklet, which supports the multidisciplinary nature of palliative care. The other booklets mainly referred to physicians and nursing staff.

### Quality appraisal

According to the final consensus mapping, all EAPC first nine domains defining optimal palliative dementia care were addressed in all the booklets, as depicted in Table 3 (Additional file 4). However, not all specific recommendations within the domains were addressed by all booklets. Recommendations with regards to `setting care goals and advance care planning’ were addressed the least, especially in the Canadian and Italian booklet, while the Irish booklet addressed some of the specific recommendations. Supporting people with mild dementia in advance care planning (recommendation 3.4) was not mentioned in any of the booklets, as all booklets described the advanced stages of dementia since the booklets are positioned at the end of life, where decision making capacity may be limited. Recommendations that were also not addressed by any of the booklets related to `Continuity of care’ (having a central care coordinator and appropriate information transfer between healthcare professionals) and to `Optimal treatment’ (interdisciplinary consultation between dementia and palliative care specialists).

The Czech booklet was the only booklet that addressed recommendation 2.5 about previously expressed preferences regarding place of care (domain 2: Person-centered care). An explicit statement about avoiding the use of restraints (recommendation 6.3, domain: Avoiding burdensome treatment) was found only in the Irish Booklet.

Based on our overall findings, we present guidance statements regarding the contents of informational booklets for family caregivers about dementia and palliative care [31] in Box 2 (Additional file 5). This may inform future updates or wider adoption of the booklets and support the development of other educational materials for family caregivers in this area.

## Discussion

The Comfort Care Booklet provides family caregivers with information concerning the trajectory of advanced dementia and a palliative approach to care. In this paper, we compared Comfort Care Booklets across six jurisdictions that were developed between 2005 and 2021. One of the most striking differences between the booklets was the distinction between the UK booklet and the original Canadian booklet. The UK booklet has been under extensive review in practice by various stakeholders since 2014, originally used in Northern Ireland, it was adapted for broader application in the UK between 2019 and 2021. In the Irish and updated Canadian booklets, the involvement of family caregivers over the last year was evident from the addition of sections that engaged family caregivers, stipulating their role in providing comfort care. Interesting in this respect is the addition of a new section on spirituality for the UK, Irish and updated Canadian booklets. This addition could thus reflect increasing awareness for spiritual care as a key component in palliative care [32]. Also, it is likely that the dominant ideology in the stakeholders’ jurisdiction and the greater representation of stakeholders involved, healthcare professionals or family caregivers, influenced topics to be included in the booklets. These findings highlight the need to involve stakeholders and have appropriate levels of representation in the development and evaluation of family and patient educational materials [16] and to be transparent in reporting the process.

In addition to the impact of stakeholder involvement, sociocultural differences emerged too. End-of-life decision making and disclosing prognostic information are both significantly influenced by socio-cultural factors [33, 34]. The UK booklet was more positive about prognosis and did not include many medical details or explicit messages, as one of the developers stated: “we tend not to talk about death”. The aim of the booklet was therefore to inform family caregivers without causing distress. In contrast, the Czech booklet was less positive about prognosis and included more detailed information and explicit messages. The historically strong paternalistic culture in the Czech health care is reported to be a barrier for patient engagement [35]; although health care regulations recognize this, reform is in progress to be more inclusive of patient autonomy. The primary aim of the booklet was thus to inform and prepare family caregivers to stimulate family caregiver engagement.

Differences in legal contexts between jurisdictions were further apparent in the status of best interests and patient autonomy or previously expressed wishes in medical decision making, and the extent to which family was involved in shared decision making. While the Czech and Irish booklet emphasized best interest decision making informed by living wills and advance directives, the updated Canadian booklet did not refer to advance directives as this term is not consistent within the legal frameworks for all Canadian provinces. Differences in legislation [34] and interpretation of decision-making processes [35, 36] are therefore important to consider when providing information about end-of-life decision making.

Finally, differences over time were apparent from our analysis. The evidence base for advance care planning for people with dementia has been growing [37]. While hardly present in most of the booklets, the recent Irish booklet contained information about end-of-life care planning to ensure that any decision making is aligned to the person’s previously stated will and preference. The updated Canadian booklet included information about Medical Assistance in Dying, while the original version referred to an illegal status of euthanasia. In addition, the text was gender-neutral and did not include male pronouns. The UK booklet had removed information about tube feeding due of the wider consensus on tube feeding being inappropriate for people with dementia at the end of life; this could reflect developments in public perception making such a statement obsolete [18].

Compared with a systematic review that mapped the components of palliative care interventions according to the EAPC domains [25], the outcome of our mapping was different. The systematic review found that interventions hardly addressed ‘applicability of palliative care’. Further, ‘prognostication and timely recognition of dying’, ‘avoiding overly aggressive, burdensome or futile treatment’ and ‘setting care goals and advance care planning’ were not always included in interventions. The Comfort Care Booklets addressed all these domains as they formed the key message of the information, except for ‘setting care goals and advance care planning’. Possibly, more information about end-of-life care planning practice could be included in future editions; advance care planning that includes the person with dementia needs to be addressed at earlier disease stages.

A strength of this study is that this cross-national comparison not only focuses on different cultures, but also captured some key developments over time. This is also a limitation of this study that compared the booklets at one point in time, and we propose to review and update information materials regularly to adopt socio-cultural and evidence-base developments. Intervals for updating the booklets should be determined by developments in evidence and public perception [17, 18]. Furthermore, although both English speaking/Northern European cultures and Mediterranean/Eastern European cultures were included in our analysis [33], our study primarily focused on western documents that were all based on an original Canadian piece and does not provide information about possible issues to consider for documents in other cultures.

## Conclusions

In conclusion, the Comfort Care Booklet covers all domains of good-quality palliative care for older people with dementia [2], but more attention for end-of-life care planning and spirituality is required. We present guidance statements regarding family information. When developing informational materials that are appropriate for the local context, it is important to consider the legal and socio-cultural environment and developments over time. We also recommend stakeholder involvement throughout the development process, end-users in particular.

## Supplementary Information

**Additional file 1:** **Box 1.** Protocol for mapping the Comfort Care Booklets’ contents against the EAPC framework

**Additional file 2: Table S1.** Summary of Comfort Care Booklet development process

**Additional file 3: Table S2.** Themes of textual revisions across the booklets with example quotes

**Additional file 4: Table S3.** Outcome of the EAPC framework mapping: recommendations addressed from domains 1-9

**Additional file 5:** **Box 2.** Guidance statements

**Additional file 6.** Full list of mySupport study group

## Data Availability

The datasets used and/or analysed during the current study are available from the corresponding author on reasonable request.

## Abbreviations

CPR: Cardiopulmonary resuscitation
EAPC: European Association for Palliative Care
WHO: World Health Organisation
