# Supplementary material for: Guidance for family about comfort care in dementia: a comparison of an educational booklet adopted in six jurisdictions over a 15 year timespan
Source: BMC Palliat Care. 2022 May 17;21:76. doi: 10.1186/s12904-022-00962-z (PMC9112535; doi:10.1186/s12904-022-00962-z)
Supplement: Supplementary file 1 — Additional file 1: Box 1. Protocol for mapping the Comfort Care Booklets’ contents against the EAPC framework [file 12904_2022_962_MOESM1_ESM.docx]

**Box 1. Protocol for mapping the Comfort Care Booklets’ contents against the EAPC framework**

| **To be performed by two individuals independently:**   1. **Read the Comfort Care Booklet** 2. **Per section, assess if and which recommendation(s) of the first 9 EAPC domains is addressed, including the explanatory text [26]***   **To be performed in a consensus discussion between the two individuals:**   1. **Compare and discuss the mappings to reach consensus, using the following criteria:**  - **The text addressed the EAPC statement as found in the *recommendation and/or the* *explanatory text*** - **The text addressed the EAPC statement *explicitly*, a statement that is implied is not specifically addressed** - **The *context* of the statement may differ between the text in the Comfort Care Booklet and the EAPC framework (that is: the EAPC framework states that family caregivers need explanation without providing detail and the Comfort Care Booklet directly provides the explanation)**   ***The first 9 EAPC domains and 47 recommendations:**  Domain 1. Applicability of palliative care   - 1.1 Dementia can realistically be regarded as a terminal condition. It can also be characterized as a chronic disease or, in connection with particular aspects, as a geriatric problem. However, recognizing its eventual terminal nature is the basis for anticipating future problems and an impetus to the provision of adequate palliative care. - 1.2 Improving quality of life, maintaining function and maximizing comfort, which are also goals of palliative care, can be considered appropriate in dementia throughout the disease trajectory, with the emphasis on particular goals changing over time. - 1.3 Palliative care for dementia should be conceived as having two aspects. The baseline is a palliative care approach. For patients with complex problems, specialist palliative care should be available. - 1.4 A palliative care approach refers to all treatment and care in dementia, including adequate treatment of behavioural and psychological symptoms of dementia, comorbid diseases, and (inter- or concurrent) health problems.   Domain 2. Person-centred care, communication and shared decision making   - 2.1 Perceived problems in caring for a patient with dementia should be viewed from the patient’s perspective, applying the concept of person-centred care. - 2.2 Shared decision making includes the patient and family caregiver as partners and is an appealing model that should be aimed for. - 2.3 The health care team should ask for and address families’ and patients’ information needs on the course of the dementia trajectory, palliative care and involvement in care. - 2.4 Responding to the patient’s and family’s specific and varying needs throughout the disease trajectory is paramount. - 2.5 Current or previously expressed preferences with regard to place of care should be honoured as a principle, but best interest, safety and family caregiver burden issues should also be given weight in decisions on place of care. - 2.6 Within the multidisciplinary team, patient and family issues should be discussed on a regular basis.   Domain 3. Setting care goals and advance planning   - 3.1 Prioritizing of explicit global care goals helps guide care and evaluate its appropriateness. - 3.2 Anticipating progression of the disease, advance care planning is proactive. This implies it should start as soon as the diagnosis is made, when the patient can still be actively involved and patient preferences, values, needs and beliefs can be elicited. - 3.3 Formats of advance care plans may vary in terms of preferences, the amount of detail required, and what is available in the specific setting for the individual. - 3.4 In mild dementia, people need support in planning for the future. - 3.5 In more severe dementia and when death approaches, the patient’s best interest may be increasingly served with a primary goal of maximization of comfort. - 3.6 Advance care planning is a process, and plans should be revisited with patient and family on a regular basis and following any significant change in health condition. - 3.7 Care plans should be documented and stored in a way that permits access to all disciplines involved in any stage and through transfers.   Domain 4. Continuity of care   - 4.1 Care should be continuous; there should be no interruption even with transfer. - 4.2 Continuous care refers to care provided by all disciplines. - 4.3 All patients should benefit from the early appointment of a central coordinator from within their care team. - 4.4 Transfers between settings require communication on care plans between former and new professional caregivers and patient and families.   Domain 5. Prognostication and timely recognition of dying   - 5.1 Timely discussion of the terminal nature of the disease may enhance families’ and patients’ feelings of preparedness for the future. - 5.2 Prognostication in dementia is challenging and mortality cannot be predicted accurately. However, combining clinical judgement and tools for mortality predictions can provide an indication which may facilitate discussion of prognosis.   Domain 6. Avoiding overly aggressive, burdensome or futile treatment   - 6.1 Transfer to the hospital and the associated risks and benefits should be considered prudently in relation to the care goals and taking into account also the stage of the dementia. - 6.2 Medication for chronic conditions and comorbid diseases should be reviewed regularly in light of care goals, estimated life expectancy, and the effects and side effects of treatment. - 6.3 Restraints should be avoided whenever possible. - 6.4 Hydration, preferably subcutaneous, may be provided if appropriate, such as in case of infection; it is inappropriate in the dying phase (*only moderate consensus*). - 6.5 Permanent enteral tube nutrition may not be beneficial and should as a rule be avoided in dementia; skilful hand feeding is preferred (*only moderate consensus*). - 6.6 Antibiotics may be appropriate in treating infections with the goal of increasing comfort by alleviating the symptoms of infection. Life-prolonging effects need to be considered, especially in case of treatment decisions around pneumonia.   Domain 7. Optimal treatment of symptoms and providing comfort   - 7.1 A holistic approach to treatment of symptoms is paramount because symptoms occur frequently and may be interrelated, or expressed differently (e.g., when pain is expressed as agitation). - 7.2 Distinguishing between sources of discomfort (e.g., pain or being cold) in severe dementia is facilitated by integrating views of more caregivers. - 7.3 Tools to assess pain, discomfort and behaviour should be used for screening and monitoring of patients with moderate and severe dementia, evaluating effectiveness of interventions. - 7.4 Both non-pharmacological and pharmacological treatment of physical symptoms, challenging behaviour or discomfort should be pursued as needed. - 7.5 Nursing care is very important to ensure comfort in patients near death. - 7.6 Specialist palliative care teams may support staff in long-term care settings in dealing with specific symptoms, while maintaining continuity of care. In managing behavioural symptoms, however, palliative care teams may need additional dementia care specialist expertise.   Domain 8. Psychosocial and spiritual support   - 8.1 In mild dementia, as also in the later stages, patients may be aware of their condition, and patients and families may need emotional support. - 8.2 Spiritual caregiving in dementia should include at least assessment of religious affiliation and involvement, sources of support and spiritual well being; in addition, referral to experienced spiritual counsellors such as those working in nursing homes may be appropriate. - 8.3 Religious activities, such as rituals, songs, and services may help the patient because these may be recognized even in severe dementia. - 8.4 For dying people, a comfortable environment is desirable.   Domain 9. Family care and involvement   - 9.1 Families may suffer from caregiver burden, may struggle to combine caring with their other duties and may need social support. - 9.2 Families may need support throughout the trajectory, but especially upon diagnosis, when dealing with challenging behaviour, with health problems, with institutionalization, with a major decline in health and when death is near. - 9.3 Families need education regarding the progressive course of the dementia and (palliative care) treatment options; this should be a continuous process addressing specific needs in different stages, examining family receptiveness. - 9.4 Family involvement may be encouraged; many families may wish to be involved in care even when the patient is admitted to an institution providing long-term care. - 9.5 Families need support in their new role as (future) proxy decision maker. - 9.6 Professional caregivers should have an understanding of families’ needs related to suffering from chronic or prolonged grief through the various stages, and with evident decline. - 9.7 Bereavement support should be offered. - 9.8 Following the death of the patient, family members should be allowed adequate time to adjust after often a long period of caring for the patient. |
| --- |
