# Supplementary material for: Guidance for family about comfort care in dementia: a comparison of an educational booklet adopted in six jurisdictions over a 15 year timespan
Source: BMC Palliat Care. 2022 May 17;21:76. doi: 10.1186/s12904-022-00962-z (PMC9112535; doi:10.1186/s12904-022-00962-z)
Supplement: Supplementary file 2 — Additional file 2: Table S1. Summary of Comfort Care Booklet development process [file 12904_2022_962_MOESM2_ESM.docx]

**Table 1: Summary of comfort care booklet development process**

| **Location, ed (year)** | **Based on** | **Stakeholder involvement**  **[type of involvement]*** | **Most influence on revisions** | **Focus of revisions**  **[derived from interview]** |
| --- | --- | --- | --- | --- |
| *CA, 1 (2005)* | *Literature;*  *[28]* | *Researchers / Healthcare professionals;*  *current and bereaved family caregivers [undefined involvement]* |  |  |
| **IT, 1 (2008)** | CA, 1 (2005) | Researchers; ethicists, physicians (LTC), nurses (LTC), bereaved family caregivers  [targeted consultation] | ? | Layout (showing Italian setting), legal context of shared decision making and euthanasia [14] |
| **NL, 2 (2011)** | CA, 1 (2005) | Researchers; ethicists, physicians (LTC), nurses (LTC), bereaved family caregivers  [targeted consultation] | LTC physicians | Order of topics (and Table of contents), legal context of shared decision making and euthanasia, treatment considerations [14] |
| **CZ, 1 (2017)** | CA, 1 (2005) | Researchers; nurses, geriatricians, palliative team members, physicians (hospice care, palliative care), psychologists, social care professionals, ethicist, law specialist, family caregivers  [targeted consultation] | All (multidisciplinary approach) | Adaptation to the Czech laws; the role of family in shared decision making  [derived from interview] |
| **IE, 1 (2020)** | UK, 2 (2017) | Researchers; GP, geriatricians, speech and language therapist, nurses, family caregivers [collaboration and co-production] | GP, geriatricians | **Legal context of shared decision making; focus on end-of-life care planning; addition of family involvement**  **[derived from interview]** |
| **UK, 3 (2021)** | CA, 1 (2005) | Researchers; geriatricians, nurses (dementia, palliative care), nursing home manager, facilitator (oncology and palliative care), hospice consultant, Alzheimer Society consultant, service user, policy officers, family caregivers  [embedded consultation] | Facilitator (oncology and palliative care), hospice consultant, Alzheimer Society | **Deletion of not-applicable material (PEG, euthanasia); addition of a section on spirituality; use of softer language and layout to prevent distress**  **[derived from interview]** |
| **CA, 2 (2021)** | CA, 1 (2005);  UK, 2 (2017) | Researchers; care director (LTC), nurses (LTC), family caregivers  [undefined involvement] | Family caregivers | **Additions of sections on spirituality, Medical Assistance in Dying, oral hygiene and mouth care; use of inclusive language and (shared decision making) terminology in accordance with current legal framework**  [derived from interview] |

CA: Canada, IT: Italy, NL: the Netherlands, CZ: the Czech Republic, UK: the United Kingdom, IE: Ireland, LTC: Long Term Care

*Undefined involvement: stakeholders act as participants/respondents/research subjects **in a study leading up to the development of the booklet, thus were indirectly involved**; Targeted consultation: stakeholders are approached to provide feedback/comments; Embedded consultation: stakeholders are regularly consulted throughout the process; Collaboration and co-production: stakeholders are part of the team and contribute to key decisions; User-led research: stakeholders take lead in designing and conducting research [27]
