# Supplementary material for: Guidance for family about comfort care in dementia: a comparison of an educational booklet adopted in six jurisdictions over a 15 year timespan
Source: BMC Palliat Care. 2022 May 17;21:76. doi: 10.1186/s12904-022-00962-z (PMC9112535; doi:10.1186/s12904-022-00962-z)
Supplement: Supplementary file 3 — Additional file 3: Table S2. Themes of textual revisions across the booklets with example quotes [file 12904_2022_962_MOESM3_ESM.docx]

**Table 2. Themes of textual revisions across the booklets with example quotes**

| **Theme** | **Example quote (identifier, source)** |
| --- | --- |
| Typology of treatments and symptoms at the end of life | UK editor about the deletion of the section on tube feeding treatment:  *In the UK, there was a PEG-tube plead. So some of the carers were a bit confused about why [information on tube feeding] was in the book, because they then said, you know: can we have that? Or: I don’t actually want that to happen. So our facilitator felt it be better to not include that part of it, because it wasn’t standard practice in the nursing homes.* (A, interview) |
| Patient rights and wishes | Canada editor (of updated Canadian booklet) about the adaptions in terminology referring to the status of the family caregiver in decision making:  *(…) we have this very strict legal framework (..) and there’s a lot of emphasis right now within our palliative care organizations to make sure that all of our advance care planning, goals of care programs and educational materials are compliant with that legal framework. (…) And then we have consent as well. So for any kind of treatment, you have to have consent from families. So that’s why we had to change the language around values and goals and wishes and then treatment plans (…).* (B, interview) |
| Typology of decisions at the end of life | Original Canadian text, with section about the moral dilemma underlying treatment decisions excluded in Italian version in **bold**:  *(…) The doctor can prescribe an antibiotic if the individual develops a fever and if it is judged, from a medical standpoint, that the individual has pneumonia. However, as mentioned earlier, the chance of recovering from pneumonia in the advanced stages of these types of neurological diseases is limited, and the possibility of this problem re-occuring shortly thereafter is elevated.*  ***William Osler, a well-known early 20th-century doctor, said that pneu­monia may very well be the older person’s best friend because it can bring suffering to an end. That is why many doctors prefer to abstain from prescribing antibiotics for pneumonia at the end of life, and select a palliative care approach such as the one described further on in this document****.*  *Each situation is evaluated on a case-by-case basis. (…)* (C, comfort care booklets) |
| Indirect or explicit messages | Original Canadian version:  *People who are lucid and who are suffering from cancer or degener­ative, neurological diseases say that the feeling of thirst and hunger is not common near the end of life. Most patients refuse all or even small amounts of food that are offered to them and say that they feel a sensation of dryness in the mouth*.  Netherlands version with added explicit message about the dying phase:  *Patients with dementia often gradually start drinking or eating less. The body adapts to this, which makes the patient appear not to be hungry or thirsty. Patients barely want to eat or drink in the final phase. A well-known saying is: ‘people do not die because they do not eat and drink; they do not eat and drink because they are dying’. (…)* (D, comfort care booklets) |
| More or less positive about prognosis | Original Canadian introduction, first paragraph:  *This guide is intended for caregivers of a per­son whose health has been severely affected by Alzheimer’s disease or by another type of degenerative disease of the brain, such as Parkinson’s disease, the effects of multiple strokes, or even certain forms of multiple sclerosis.*  Czech Republic version introduction, first paragraph with added section implying a less positive perspective on prognosis:  *This guide is intended for those who care for people with Alzheimer's disease or any other type of brain disease, such as Parkinson's disease or vascular dementia. All these diseases can cause dementia syndrome. A disease causing dementia is a life-limiting disease in its effects. Everyone knows someone who has been living with dementia for ten or even fifteen or more years. However, the most common survival time from the diagnosis of dementia is four to five years. Some people also have other serious illnesses at the time of diagnosis of dementia, such as heart disease, while others develop cancer. Others, however, die because dementia reduces their life expectancy. This guide focuses on the challenging times of the very advanced and final stages of the disease.* (E, comfort care booklets) |
| Relationship among healthcare professionals and family caregivers | Irish editor about the addition of sections for family caregivers as partners in care:  *Yes, like I think, in particularly, some of the information was very kind of, kind of soft touch in one sense, and it was kind of ‘nice to know’-material, you know. But we weren’t sure exactly what the family could learn from it, in one sense. So that’s why we divided sections up in two: ‘What the healthcare professional does’ and ‘What you can do as a family’. Kind of around the oral care in particular, you know, so it’s kind of giving them something to do, they feel useful, but it’s also informing them this is what to expect the healthcare professional is going to be doing, you know.* (F, interview) |
