# Supplementary material for: Guidance for family about comfort care in dementia: a comparison of an educational booklet adopted in six jurisdictions over a 15 year timespan
Source: BMC Palliat Care. 2022 May 17;21:76. doi: 10.1186/s12904-022-00962-z (PMC9112535; doi:10.1186/s12904-022-00962-z)
Supplement: Supplementary file 4 — Additional file 4: Table S3. Outcome of the EAPC framework mapping: recommendations addressed from domains 1-9 [file 12904_2022_962_MOESM4_ESM.docx]

**Table 3: Outcome of the EAPC Framework mapping: recommendations* addressed from domains 1-9**

| **Location, ed (year)** | Domain 1  Applicability of palliative care  (1.1-1.4) | Domain 2  Person-centered care, communication and shared decision making  (2.1-2.6) | Domain 3  Setting care goals and advance care planning  (3.1-3.7) | Domain 4  Continuity of care  (4.1-4.4) | Domain 5  Prognostication and timely recognition of dying  (5.1-5.2) | Domain 6  Avoiding overly aggressive, burdensome or futile treatment  (6.1-6.6) | Domain 7  Optimal treatment of symptoms and providing comfort  (7.1-7.6) | Domain 8  Psychosocial and spiritual support  (8.1-8.4) | Domain 9  Family care and involvement  (9.1-9.8) |
| --- | --- | --- | --- | --- | --- | --- | --- | --- | --- |
| **IT, 1**  **(2008)** | 1.1-1.4 | 2.1-2.4, 2.6 | 3.5 | 4.1-4.2 | 5.2 | 6.1-6.2, 6.4-6.6 | 7.1-7.5 | 8.2 | 9.2-9.5, 9.7-9.8 |
| **NL, 2 (2011)** | 1.1-1.2, 1.4 | 2.1-2.4, 2.6 | 3.3, 3.5 | 4.1-4.2 | 5.2 | 6.1-6.2, 6.4-6.6 | 7.1-7.5 | 8.2-8.4 | 9.2-9.5, 9.7 |
| **CZ, 1 (2017)** | 1.1-1.2, 1.4 | 2.1-2.5 | 3.2, 3.3, 3.5 | 4.1-4.2 | 5.2 | 6.1-6.2, 6.4-6.6 | 7.1-7.5 | 8.2, 8.4 | 9.2-9.5, 9.7-9.8 |
| **IE, 1 (2020)** | 1.1-1.4 | 2.1-2.4, 2.6 | 3.1-3.3, 3.5-3.7 | 4.1 | 5.1-5.2 | 6.1-6.6 | 7.1-7.5 | 8.1-8.3 | 9.2-9.7 |
| **UK, 3 (2021)** | 1.1-1.2, 1.4 | 2.1-2.4, 2.6 | 3.2, 3.5, 3.7 | 4.1-4.2 | 5.1-5.2 | 6.1-6.2, 6.4-6.6 | 7.1-7.5 | 8.1-8.4 | 9.1-9.5, 9.7-9.8 |
| **CA, 2 (2021)** | 1.1-1.2, 1.4 | 2.1-2.4, 2.6 | 3.5 | 4.1-4.2 | 5.1-5.2 | 6.1-6.2, 6.4-6.6 | 7.1, 7.3-7.5 | 8.1-8.4 | 9.2-9.5, 9.7 |

*Numbers in the table refer to recommendation numbers as listed in the EAPC Framework [2]
