# Supplementary material for: Guidance for family about comfort care in dementia: a comparison of an educational booklet adopted in six jurisdictions over a 15 year timespan
Source: BMC Palliat Care. 2022 May 17;21:76. doi: 10.1186/s12904-022-00962-z (PMC9112535; doi:10.1186/s12904-022-00962-z)
Supplement: Supplementary file 5 — Additional file 5: Box 2. Guidance statements [file 12904_2022_962_MOESM5_ESM.docx]

**Box 2: Guidance statements**

| Core statements of information for family caregivers about dementia and comfort care  *Information for family caregivers of nursing home residents with advanced dementia should* *advise a palliative approach and cover*  **the course of the dementia and expected complications:**   1. Advanced dementia should be considered a terminal illness with the majority of patients dying from nutrition/hydration or infectious problems, especially pneumonia.*   **the decision-making process:**   1. In decisions regarding whether or not to use life-prolonging therapy in advanced dementia, the ideal decision-making process is to reach a consensus between the physician, the substitute decision maker and other significant relatives or friends of the patient.* 2. In the decision-making process, the medical options that are most suited to the patient’s best interests (e.g. according to patient values and written or verbal advanced directives) should be strived for and options that would not presumably be acceptable to the patient should be refused.**   **symptom management:**   1. Use of parenteral hydration (IV or hypodermoclysis) may be helpful in selected cases but can also contribute to discomfort.** 2. A patient who no longer eats and drinks will generally not experience discomfort if there is adequate mouth care.** 3. When ‘comfort care without life prolongation’ is the goal of care, antibiotics can be withheld and treatment will then aim at symptom control.* 4. Prescription of opioids may be necessary to control pain or breathing difficulties to relieve the patient.**   **avoiding burdensome treatment:**   1. Cardiopulmonary resuscitation is not recommended in advanced dementia because it can harm the patient and has very little chance of success.* 2. Hospital transfer of the patient with advanced dementia should be exceptional e.g. only to provide comfort by technical means not available in the nursing home (such as hip fracture surgery).*   **dying and grief:**   1. It is difficult to estimate the moment of death, family caregivers should be accommodated to stay with the patient during the end of life. 2. Family caregivers should be offered bereavement support to cope with feelings of grief, relief, anger, loneliness, exhaustion and guilt.   *In addition, information needs to be provided about*  **advance care planning:**   1. Prioritizing explicit care goals, if possible together with the patient, should start as soon as possible and should be evaluated regularly to help guide care.   **spiritual care:**   1. Patients and family caregivers should be offered spiritual care, religious activities or support by spiritual counsellors for psychosocial and spiritual support.   Flexible elements of information for family caregivers about dementia and comfort care  *Information for family caregivers of nursing home residents with advanced dementia that requires adaption to the local context includes:*   - Information about withholding/withdrawing of artificial nutrition/hydration - Information about the roles and responsibilities of the patient, substitute decision makers, healthcare professionals and others involved in medical decision making and caregiving - Information about life terminating treatments - Information about (palliative) sedation to relieve anxiety or agitation - The use of medical details or terminology - The expression of moral considerations underlying treatment decisions - The use of imagery and tone of voice   Process of developing information for family caregivers about dementia and comfort care  *Information for family caregivers of nursing home residents with advanced dementia should be developed*   1. with the involvement of stakeholders (family caregivers, healthcare professionals, ethicists, law specialists) in constructing and evaluating the content 2. continuously, that is: regularly updated to keep information in line with current practice and public perception 3. in a manner that is sensitive to the legal and socio-cultural context 4. with a clear reporting about the process and people involved |
| --- |

** These statements are retained from the core statements by Arcand et al. [31]*

***These statements are adapted from the core statements by Arcand et al. [31]*
