# Supplementary material for: Guidance for family about comfort care in dementia: a comparison of an educational booklet adopted in six jurisdictions over a 15 year timespan
Source: BMC Palliat Care. 2022 May 17;21:76. doi: 10.1186/s12904-022-00962-z (PMC9112535; doi:10.1186/s12904-022-00962-z)
Supplement: Supplementary file 6 — Additional file 6. Full list of mySupport study group [file 12904_2022_962_MOESM6_ESM.docx]

**mySupport study group**

mySupport study group members:

Marcel Arcand^8^, Jackson Hagner^11^, Danielle Just^4^, Sharon Kaasalainen^4^, Tamara Sussman^11^, Martin Loucka^5^, Hana Vankova^5^, Karolina Vlckova^5^, Ladislav Volicer^7^, Marco Clari^6^, Paola Di Giulio^6^, Silvia Gonella^6^, Laura Simionato^12^, Wilco Achterberg^1^, Laura Bavelaar^1^, Jenny T. van der Steen^1,9^* Mandy Visser^1^, Catherine Buckley^13^, Nicola Cornally^3^, Serena Fitzgerald^3^, Tony Foley^14^, Siobhan Fox^15^, Irene Hartigan^3^, Dominika Lisiecka^16^, Ronan O’Caoimh^15^, Selena O’Connell^17,18^, Catherine Sweeney^19^, Suzanne Timmons^15^, Kevin Brazil^10^, Christine Brown Wilson^10^, Gillian Carter^10^, Emily Cousins^20^, Kay De Vries^20^, Josie Dixon^21^, Andrew Harding^22^, Karen Harrison Dening^23^, Catherine Henderson^21^, Adrienne McCann^2^, Sophie Morris^10^, Nancy Preston^22^, Catherine Walshe^22^

^1^ Department of Public Health and Primary Care, Leiden University Medical Center, Leiden, the Netherlands. ^2^ Innovation Value Institute, Maynooth University/Age Friendly Ireland , Ireland. ^3^ Catherine McAuley School of Nursing and Midwifery, University College Cork, Cork, Ireland. ^4^ School of Nursing, McMaster University, Ontario, Canada. ^5^ Center for Palliative Care, Prague, and Third Faculty of Medicine, Charles University, the Czech Republic. ^6^ Department of Sciences of Public Health and Pediatrics, Turin University, Milan, Italy. ^7^ School of Aging Studies, University of South Florida, Tampa, FL, USA. ^8^ Department of Family Medicine, University of Sherbrooke, Sherbrooke, QC, Canada. ^9^ Department of Primary and Community Care, Radboud university medical center, Nijmegen, the Netherlands. ^10^ School of Nursing and Midwifery, Queen’s University Belfast, Belfast, Northern Ireland, the UK. ^11^ School of Social Work, McGill University, Montreal, Canada. ^12^ Department of Clinical and Biological Sciences, University of Torino, Torino, Italy. ^13^ Northridge House Education & Research Centre, St. Lukes Home, Cork, Ireland. ^14^ Department of General Practice, University College Cork, Cork, Ireland. ^15^ Centre for Gerontology and Rehabilitation, School of Medicine, University College Cork, Cork, Ireland. ^16^ Department of Nursing and Healthcare Sciences, School of Health and Social Sciences, Munster Technological University, Tralee, Ireland. ^17^ National Suicide Research Foundation, University College Cork, Cork, Ireland. ^18^ School of Public Health, College of Medicine and Health, University College Cork, Cork, Ireland. ^19^ Department of Medicine, University College Cork, Cork, Ireland. ^20^ School of Nursing and Midwifery, De Montfort University, Leicester, the UK. ^21^ Care Policy and Evaluation Centre, London School of Economics and Political Science, London, the UK. ^22^ Department of Health Research, Lancaster University, Lancaster, the UK. ^23^ Dementia UK, London, the UK.
